# Supplementary material for: Ectomycorrhizal Fungi Modulate Pedunculate Oak’s Heat Stress Responses through the Alternation of Polyamines, Phenolics, and Osmotica Content
Source: Plants (Basel). 2022 Dec 3;11(23):3360. doi: 10.3390/plants11233360 (PMC9736408; doi:10.3390/plants11233360)
Supplement: Supplementary file 1 [file plants-11-03360-s001.zip › plants-2027420-supplementary.pdf]

Title
Ectomycorrhizal fungi modulate pedunculate oak's heat stress responses through the alternation of polyamines, phenolics, and osmotic content

Supplementary material

Table S1. A two-way ANOVA results on variable (ECM, HS ECMxHS) effects on inspected parameters.

**Parameters**		Mycorriza			Temperature			Mycorriza:Temperature		
		**F**	***p<.05***	***p***	**F**	***p<.05***	***p***	**F**	***p<.05***	***p***
**Osmolytes**	Glycine betaine **(GB)**	0.75	ns	0.41	16.81	*	3×10^{-3}	28.17	*	7.22×10^{-4}
	Free proline **(PRO)**	4.82	ns	0.59	25.44	*	9.97×10^{-4}	0.14	ns	0.72
	Total polyamine content **(Pas)**	9.53	*	0.02	18.64	*	3×10^{-3}	3.60	ns	0.94
	Putrescine **(PUT)**	319.48	*	9.84×10^{-8}	15.03	*	0.01	4.70	ns	0.06
	Spermine **(SPM)**	4.56	ns	0.07	12.43	*	0.01	4.49	ns	0.07
	Spermidine **(SPD)**	16.25	*	4×10^{-3}	15.28	*	4×10^{-3}	16.07	*	4×10^{-3}
**Antioxidant parameters**	Lipid peroxidation **(MDA)**	24.55	*	1×10^{-3}	9.22	*	0.02	0.56	ns	0.48
	Total non-protein thiols **(GSH)**	12.23	*	0.01	3.74	ns	0.09	2.54	ns	0.15
	Radical scavenger capacity (RSC) against ABTS radical **(ABTS)**	15.16	*	0.01	21.34	*	2×10^{-3}	3.82	ns	0.09
	Ferric reducing ability of extract/plasma **(FRAP)**	3.26	ns	0.11	154.48	*	1.64×10^{-6}	73.04	*	2.71×10^{-5}
	Total phenolic content **(TPC)**	1.58	ns	0.24	175.08	*	1.01×10^{-6}	70.33	ns	3.11×10^{-5}
	Total flavonoid content **(TFC)**	33.75	*	4×10^{-4}	62.89	*	4.65×10^{-5}	6.14	*	0.04
	Condensed tannins **(CT)**	0.33	ns	0.58	0.23	ns	0.65	1.72	ns	0.23
**Physiological parameters**	Relative water content **(RWC)**	3.69	ns	0.91	12.91	*	0.01	6.73	*	0.03
	Intercellular CO2 concentration **(Ci)**	18.87	*	3×10^{-3}	0.14	ns	0.71	11.58	*	0.01
	Net photosynthesis **(A)**	34.49	*	3.73×10^{-4}	1.27	ns	0.29	8.16	*	0.21
	Transpiration rate **(E)**	0.99	ns	0.76	0.34	ns	0.58	4.82	ns	0.06
	Stomatal conductance **(gs)**	0.98	ns	0.35	1.16	ns	0.31	9.91	*	0.01
	Water use efficiency **(WUE)**	16.01	*	4×10^{-3}	0.05	ns	0.84	12.56	*	0.01
**Mineral elements**	Nitrogen content **(N)**	3.17	ns	0.11	16.25	*	4×10^{-3}	0.97	ns	0.36
	Carbon content **(C)**	2.07	ns	0.19	5.42	*	0.05	0.50	ns	0.50
